# Validation of the Sexual Knowledge Picture Instrument as a diagnostic instrument for child sexual abuse: study protocol

**DOI:** 10.1136/bmjpo-2020-000799

**Published:** 2020-09-29

**Authors:** Kirsten van Ham, Sonja Brilleslijper-Kater, Hanneke van der Lee, Rick van Rijn, Hans van Goudoever, Rian Teeuw

**Affiliations:** 1 Paediatrics, Amsterdam UMC Locatie AMC, Amsterdam, The Netherlands; 2 Epidemiology, Kennisinstituut van Medisch Specialisten, Utrecht, The Netherlands; 3 Paediatric Radiology, Amsterdam UMC Locatie AMC, Amsterdam, The Netherlands

**Keywords:** child abuse, epidemiology

## Abstract

**Background:**

The consequences of child sexual abuse (CSA) can be significant and can affect short-term and long-term mental, sexual and physical health. In order to offer timely and appropriate care for the child, early recognition of CSA is necessary. The lack of specific physical and psychological signs and barriers to abuse disclosure that these young victims face makes it difficult for medical and psychological professionals to recognise and confirm CSA signs. We aimed to validate the Sexual Knowledge Picture Instrument (SKPI) as a diagnostic instrument for CSA.

**Methods and analysis:**

An observational study to quantify the intraobserver and interobserver reliability and diagnostic accuracy of the SKPI will be performed. A total of 250 subjects from three groups will be included in the study: (1) a group of suspected CSA victims, recruited from three academic paediatric hospitals; (2) a case group of (proven) victims of CSA, recruited in cooperation with the Dutch Police Vice Squad; and (3) a control group of children, recruited from preschools and primary schools. All children will be interviewed using the SKPI, and to investigate reliability, video recordings will be assessed and reassessed by the same and a different blinded rater, respectively. Within 1 year, the results of the SKPI will be compared with the conclusions from the independent child protective services or police reports. If necessary, the SKPI will be modified to improve its reliability and accuracy.

**Ethics and dissemination:**

This validation study of the SKPI is necessary for obtaining a reliable diagnostic tool, which will enable medical and psychological professionals to detect CSA in young victims at an early age and start intervention/treatment.

**Trial registration number:**

NL 50903.018.15.

**What is already known on this topic?**

- Regardless of major consequences, sexual abuse in young children often remains unrecognised.
- To date, no validated diagnostic tool is available for medical and psychological professionals in case of suspected sexual abuse in a young child.

**What this study hopes to add?**

- This is a protocol of the first clinical study aiming to develop and validate a practical diagnostic tool for child sexual abuse, the Sexual Knowledge Picture Instrument (SKPI).
- The inter-rater and intrarater reliability and diagnostic accuracy of the SKPI will be quantified, possibly enabling a more widespread dissemination.

## Introduction

Child sexual abuse (CSA) is a major problem with an estimated prevalence varying from 8% to 31% for girls and from 3% to 17% for boys.^1^ Of all victims of CSA, 25%–35% are under 7 years of age.^2^ Despite the devastating short-term, medium-term and long-term effects that sexual abuse may cause, it often remains unrecognised even into adulthood.^3 4^ Young sexually abused children often lack specific physical or psychological symptoms and experience many barriers to speak out about the abuse.^5–8^ No valid diagnostic instruments are available for medical and psychological professionals to help diagnose this type of abuse in this young population. Some parental questionnaires, such as the Child Sexual Behaviour Inventory (CSBI) and the Trauma Symptom Checklist for Young Children (TSCYC), are available.^9–13^ These lists are only of limited value as they are not capable of detecting specific CSA symptoms, and response bias may occur.^14–17^

In the early 1990s, the Sexual Knowledge Picture Instrument (SKPI) was developed to serve as a tool to assess sexual knowledge in young children.^18^ Although sexual knowledge also depends on other factors, such as family and community attitudes towards sexuality, age-inappropriate knowledge can be a sign of CSA and should be (objectively) measured by trained medical and psychological professionals.^19 20^

The SKPI is a child-friendly picture book which contains a number of illustrations about everyday routines, in addition to representation of several sexually related topics: (1) physical differences between boys and girls, (2) gender identity, (3) genitals and their functions, (4) sexual behaviour of adults, and (5) boundaries between physical intimacy and sexual acts (see online supplemental appendix 1). The tool allows a trained medical or psychological professional to ask open questions and to conduct a conversation about these topics. Comparing sexual knowledge in a group of preschool aged, non-abused and abused children, we found that non-abused children’s reactions to the questions and pictures were mostly relaxed, open-minded and unprejudiced.^21 22^ In contrast, the abused children, who seemed remarkably more reserved and stressed when interviewed, revealed less basic sexuality knowledge and showed significantly more avoidant non-verbal/emotional reactions during the interview.^21^ The SKPI additionally seemed to offer an opportunity for some abused children to speak out ‘in their own words’ by allowing them to disclose their abuse despite having a limited vocabulary.

10.1136/bmjpo-2020-000799.supp1 Supplementary data

The results supported the hypothesis that the SKPI could be of value during the assessment of suspected CSA in young children. It formed the basis of our protocol for the Picture Instrument for Child Sexual Abuse Screening (PICAS) study. The PICAS study aims to determine the reliability and diagnostic accuracy of the SKPI and to establish its validity as a CSA diagnostic tool for medical and psychological professionals to use in children who are at the developmental ages of 3–9 years old.

The original SKPI illustrations were redrawn to create a contemporary look and feel (online supplemental appendix 1). A manual was developed for medical and psychological professionals (online supplemental appendix 2) in consultation with the Dutch Police Academy and Public Prosecution Service. It contains instructions for the interviewer, a semistructured interviewing protocol developed on the basis of the 10-step principles by Thomas Lyon (an adaptation of the National Institute of Child Health and Human Development Investigative Interview Protocol), and a scoring list for verbal and non-verbal responses.^23^

10.1136/bmjpo-2020-000799.supp2 Supplementary data

## Methods and analysis

### Study design

**Figure 1:**
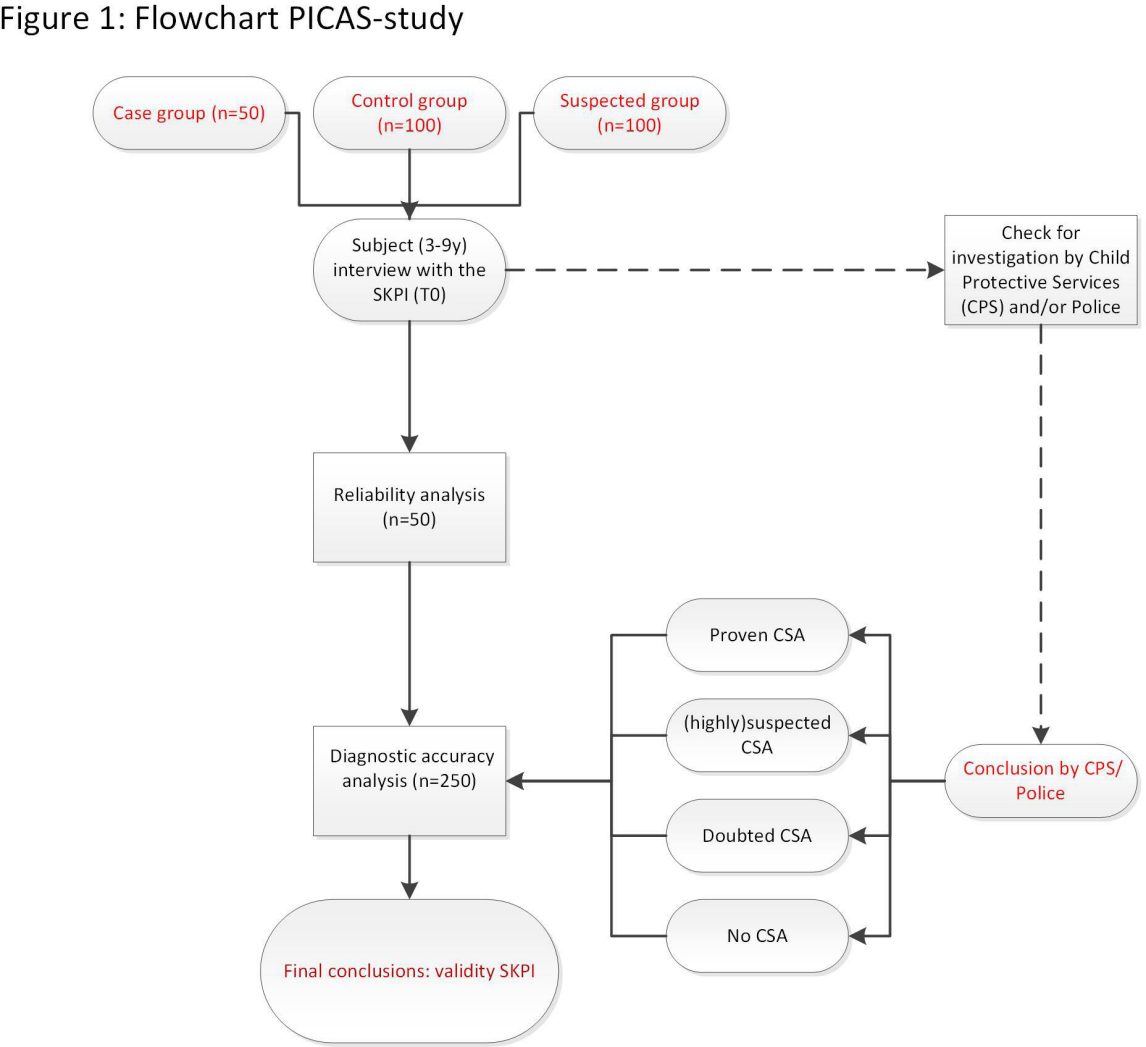
Flowchart PICAS-study. CSA, child sexual abuse; PICAS, Picture Instrument for Child Sexual Abuse Screening; SKPI, Sexual Knowledge Picture Instrument.

A total of 250 included subjects will be interviewed by a trained interviewer using the SKPI. All interviews will be video-recorded and scored afterwards. We will assess the inter- rater reliability of the SKPI by comparing rater scores from two different, independent raters. Intrarater reliability will be assessed by comparing the scores of one rater with at least 8 weeks in between to preclude remembering the SKPI. After 6–12 months, at which time an independent investigation may be finished, we will get into contact with the Dutch Child Abuse Counseling and Reporting Center (CACRC) and/or involved police officer to check for their documentation on each subject and to compare available conclusions to the SKPI results (see figure 1).

### Study population and inclusion criteria

Children aged 3–9 years old will be recruited and divided into three groups:

1. Suspected study group (n=100): the children in this group are suspected of being victims of CSA. They will be recruited after being referred to one of the specialised outpatient departments of the Emma Children’s Hospital, Amsterdam University Medical Center (UMC), the Wilhelmina Children’s Hospital, UMC Utrecht in Utrecht and the Sophia Children’s Hospital, Erasmus MC in Rotterdam, all based in the Netherlands. During the appointment, the paediatrician will inform the parents about the study and ask them whether they and their child want to participate in the study.
2. Case group (n=50): vice squad detectives within the Dutch police will inform the parents of any child who is supposed to undergo an interrogation because of highly suspected or proven CSA about this study and will ask them if they want to participate. Proven CSA victims are those subjects of whom footage (such as images and video recordings) or clear forensic evidence was traced and proven or the perpetrator has confessed or has been caught in the act of sexual abuse.
3. Control group (n=100): an information leaflet (see Appendix 3) will be given to the parents by teachers at participating (pre)schools. Based on the answers of participating parents to a questionnaire concerning previous or current suspicions of CSA in their child and by verification of involvement of CACRC with permission of the parents, CSA will be ruled out as much as possible in this control group.

10.1136/bmjpo-2020-000799.supp3 Supplementary data

In order to be eligible to participate in this study, informed consent must be provided by the parent(s)/legal guardian(s), and all subjects must meet several criteria, which will be checked and verified in the questionnaires for the parents: (1) normal cognitive development, (2) normal visual abilities and (3) no diagnosed psychiatric and/or behavioural disorder. Participation is only possible when the child has never been previously interviewed using the SKPI.

### Informed consent

The parent(s)/legal guardian(s) of all recruited subjects will be informed, both written and verbally, in detail about the study procedures and study aims. If parent(s)/legal guardian(s) have any questions concerning the study procedures, they can contact either the coordinating researcher or an external expert at any time during the course of the study. After a reflection period of at least 1 week, if they decide to participate in the study, the parent(s)/legal guardian(s) will be asked to sign an informed consent form. This form includes permission for the researchers of coded data storage according to Good Clinical Practice (GCP) guidelines to request and/or share any necessary information with the police and/or CACRC, and to make video recordings of the SKPI interview. The parent(s)/legal guardian(s) can withdraw from the study at any time without given reasons for withdrawal and without any consequences.

### Child interview with the SKPI

All study participants will undergo a structured interview with the SKPI, which will be video-recorded. Before conducting interviews, the interviewers will undergo structured training concerning the use of the SKPI and its manual.

At the start of each interview, the interviewer will instruct the child that there are no ‘wrong answers’ and to tell the interviewer whenever he or she (the child) does not understand, does not know or does not want to reveal the answer. Thereafter, the interviewer will show consecutive pictures from the SKPI and ask the corresponding open, non-suggestive questions from the manual. Video recording enables the interviewer and researchers to (re)evaluate the child’s verbal and non-verbal responses and to score each interview according to the manual. All verbal and non-verbal scoring items will be described by the scoring lists from the manual (online supplemental appendix 2). The first 50 videos are also scored by an independent researcher and a second time after at least 8 weeks by the first rater (researcher) to evaluate inter-rater and intrarater reliability, respectively.

### Questionnaires for the parents

The parent(s)/caregiver(s) of each study participant will be asked to complete three digital questionnaires (online supplemental appendix 3).

1. The first questionnaire contains questions about the child’s age, school career, languages the child speaks, ethnicity (of both parents and the child), the presence of cognitive, visual and/or hearing impairments, and confirmed/treated psychiatric disorders (such as attention deficit hyperactivity disorder and autism). Parents are asked whether any past or present social support or involvement of CACRC occurred within the family of the child and if there have ever been suspicions of CSA, other forms of child abuse and/or any behavioural problems in the child.
2. The second questionnaire is the TSCYC, a standardised and normed measurement instrument for young children who have been exposed to traumatic events.^12 13^
3. The third questionnaire, the CSBI, contains questions regarding parental observations of sexual behaviour of the child.^10 11^

It will take an estimated 35 min for the parents to fill out all three questionnaires. Data will be collected in Castor Electronic Data Capture (EDC).^24^

### Abuse status verification

After inclusion (6–12 months), the coordinating researcher will retrieve any available information from each study participant about independent investigations by CACRC and/or the police. Their final conclusion will be categorised into one of four categories: (1) proven CSA, (2) highly suspected CSA, (3) doubtful/low suspicion of CSA or (4) no suspicion of CSA. This categorisation will be used as the reference standard for the diagnostic accuracy study. The used criteria for the categorisation system were based on the classification scale for CSA from the WHO Guidelines for medicolegal care for victims of sexual violence.^25^

The procedures are shown in a flowchart (figure 1).

### Additional care of subjects

If a subject shows any signs of discomfort or psychological stress during the interview using the SKPI (indicating possible CSA or other type of traumatic events), appropriate psychological help will be offered immediately by a specialised child psychologist and a follow-up appointment at an outpatient clinic will be scheduled.

### Objection by minors or incapacitated subjects

The study adheres to the code of conduct relating to expressions of objection by minors participating in medical research as formulated by the Dutch Association for Paediatric Medicine (Nederlandse Vereniging voor Kindergeneeskunde, NVK).^26^

### Patient and public involvement

We received input from several adult CSA survivors who lived with the burdens of the abuse throughout their childhood. The aim was to carefully assess and evaluate each step of the study with them. We intend to disseminate the main results to all parents and caregivers from the included subjects, as well as these CSA survivors, and will continue seeking their involvement in the development of a tool and appropriate methods of dissemination.

### Benefit and risk assessment and group relatedness

The SKPI is a child-friendly picture book which was especially developed for the target group of young children aged 3 years and up to and including 8 years at time of inclusion. Risks associated with participation can be considered negligible, and the burden can be considered minimal.

In case any unexpected findings occur in any subject from the control group, we will inform the parents and act in accordance with the Dutch reporting code of domestic violence and child abuse and neglect.^27^

**Figure 2:**
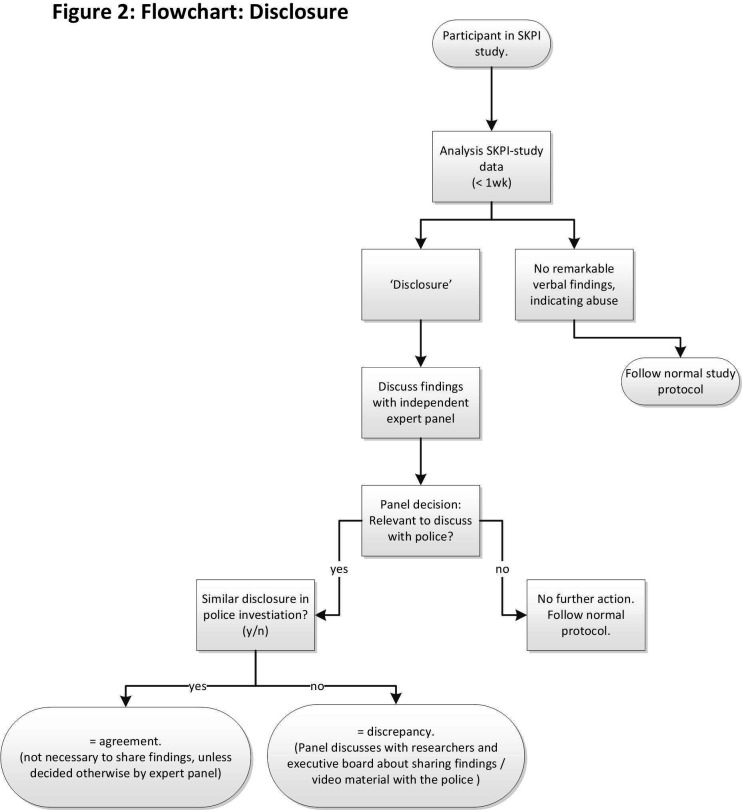
Flowchart: disclosure. SKPI, Sexual Knowledge Picture Instrument.

If unexpected findings occur during the interview with the SKPI (eg, a child who discloses being sexually abused), the interviewer will contact an expert panel in accordance with the same reporting code. Using the video material, if necessary, the experts will decide whether or not it is necessary to take additional steps in order to help the child in the best way possible (such as to verbally inform the parent(s)/legal guardian(s), other caregivers or vice detectives). If considered necessary by the experts, the research team will contact the Amsterdam UMC board to discuss whether (part of) the video material from the interview will be shared with other professionals (eg, sharing with vice detectives for subjects from the case group). The flowchart for this procedure is shown in figure 2.

### Data storage and safety

After written informed consent is obtained, all subjects will be assigned a study number. A locked key file connects this number to the subject details and is stored digitally on the Amsterdam UMC data server. This file can only be approached by the coordinating researcher and one of the local coinvestigators. The key will be destroyed after the study has been completed.

All study data will be stored according to GCP guidelines. Coded data from the subject scoring and questionnaires will be stored in Castor EDC.^24^ For privacy reasons, the video recordings will stored in a separate, locked database on the Amsterdam UMC data server in which it can only be approached by the coordinating researcher and one of the local coinvestigators. The recordings will be erased 1 year after the study’s final analysis.

### Study analysis

#### Study parameters/endpoints

The study will have two main study parameters/endpoints: (1) the SKPI’s intraresearcher and inter-researcher reliability (such as Cohen’s kappa and median kappa, IQR and limits of agreement) and (2) diagnostic accuracy (such as sensitivity, specificity and likelihood ratios).

### Statistical analysis

Study data will be analysed using SPSS V.20 or higher. Descriptive statistics (means and SD, medians and IQRs, and percentages) will be used to describe the demographic characteristics of the complete sample.

The SKPI’s scoring method yields two results: (1) sum score and (2) a red flag score. The sum score is obtained by summing all observed non-verbal behaviours for all 13 pictures in the SKPI. A total of 26 non-verbal behaviours can be scored on the form. This type of scoring means theoretically it ranges from 0 to (13×26=) 338. The red flag score contains the researcher’s overall impression from the verbal and non-verbal behaviours of the child during the interview, and those reactions will be noted by the researcher as ‘striking’ or ‘suspicious’. A red flag overrules all other information from the interview. If a red flag occurs, the test (SKPI) is considered positive, irrespective of the number score.

The intrarater and inter-rater reliability will be investigated on the video recordings of the first 50 study participants. On the level of individual items, Cohen’s kappa will be calculated to identify items that are difficult to score. Bland-Altman plots will be constructed for the summed scores and to check for systematic differences (mean difference between first and second rating) and random variation (limits of agreement). The red flag scores will be evaluated using Cohen’s kappa.

For the diagnostic accuracy study, at the start of this study, we will use a cut-off summed score of 15 points. This score will be based on the results of the earlier study, in which mean (SD) numbers of recorded behaviours of 45.9 (31.6) and 7.3 (10.9) were found in the sexually abused and non-abused groups, respectively. Based on the data generated in the current project, we will adapt this cut-off score using a receiver operating characteristic curve.

### Sample size calculation

The sample size calculation for this study was based on the book *Measurement in Medicine* by de Vet *et al*,^28^ which proposes a minimum sample size of 50 for a clinimetric validation study. By including 250 children, we aimed to obtain 95% CIs around the estimates of proportions (such as sensitivity and specificity) smaller than ±10%.

### Blinding

In the reliability study, the (second) researcher who reviews the video recordings will be blinded to the suspected abuse status or any other diagnostic information. In order to establish this set-up, the video recording of the interview will take place at the child’s home or in a neutral room in the outpatient clinic.

## Ethics and dissemination

In December 2015, the approval to start the PICAS project was obtained from the medical research ethics board of the Amsterdam UMC, location AMC. An additional approval was given by the board in 2018 for the extension of the study to a multicentre study. Authorisations to conduct the study were obtained from the UMC Utrecht and the Erasmus Medical Center in Rotterdam.

### Regulation statement

The study will be conducted according to the principles of the Declaration of Helsinki (64th WMA General Assembly, Fortaleza, Brazil, October 2013) and in accordance with the Dutch Medical Research Involving Human Subjects Act. After the PICAS study is completed, the reliability and diagnostic accuracy of the SKPI for the assessment of suspected CSA can be established. The results of the PICAS study will be published in the scientific literature, and necessary adjustments can be made for improvement of the instrument. If the SKPI is proven to be reliable and accurate, first steps can be taken to implement the SKPI in daily practice in the Netherlands and abroad. As part of the implementation strategy, a training programme with certification will be developed.
