# Supplementary material for: Validation of the Sexual Knowledge Picture Instrument as a diagnostic instrument for child sexual abuse: study protocol
Source: BMJ Paediatr Open. 2020 Sep 29;4(1):e000799. doi: 10.1136/bmjpo-2020-000799 (PMC7526291; doi:10.1136/bmjpo-2020-000799)
Supplement: Supplementary data [file bmjpo-2020-000799supp001.pdf]

Appendix I – Pictures in the Sexual Knowledge Picture Instrument (SKPI)

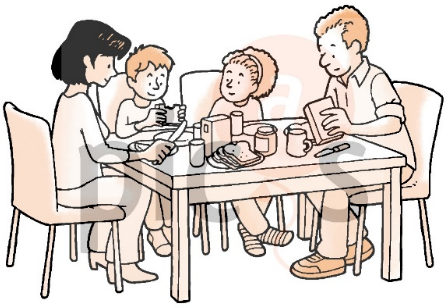

Picture 1

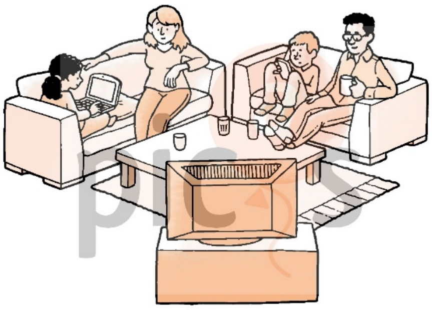

Picture 2

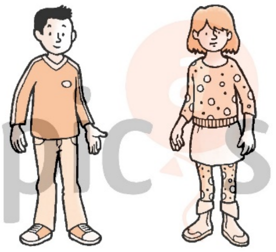

Picture 3

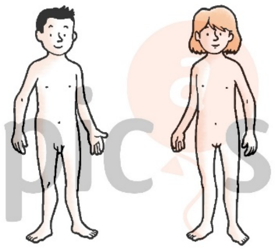

Picture 4

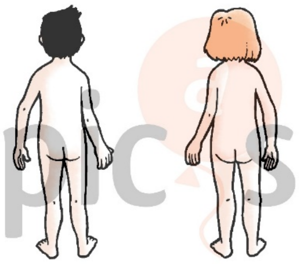

Picture 5

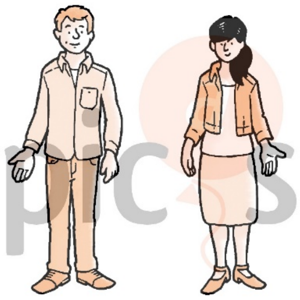

Picture 6

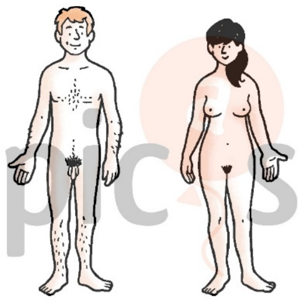

Picture 7

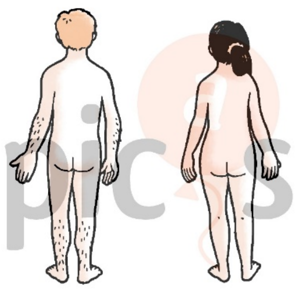

Picture 8

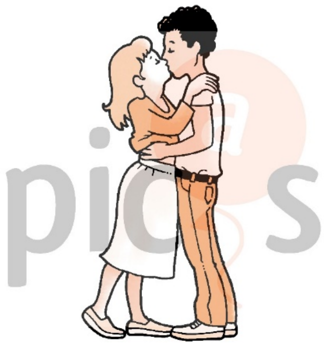

Picture 9

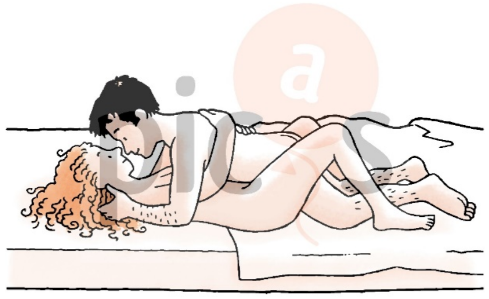

Picture 10

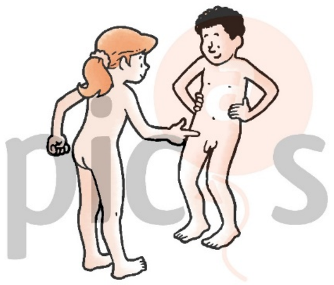

Picture 11

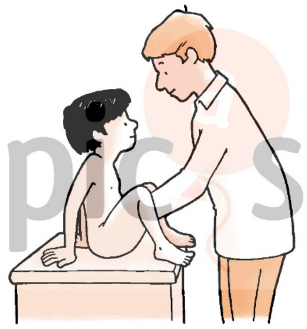

Picture 12

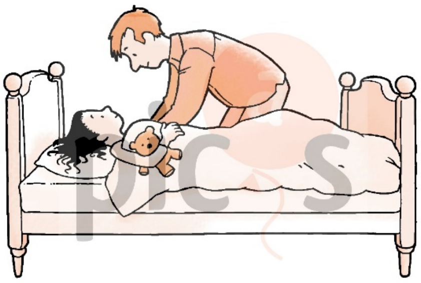

Picture 13

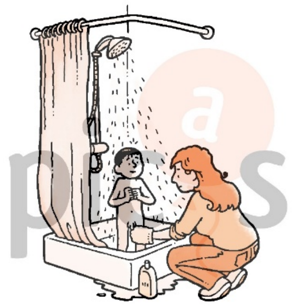

Picture 14

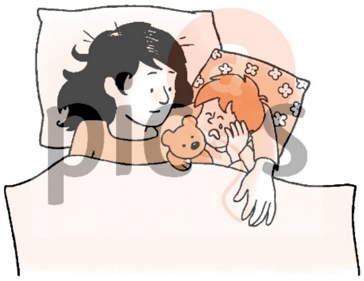

Picture 15
