# Supplementary material for: Validation of the Sexual Knowledge Picture Instrument as a diagnostic instrument for child sexual abuse: study protocol
Source: BMJ Paediatr Open. 2020 Sep 29;4(1):e000799. doi: 10.1136/bmjpo-2020-000799 (PMC7526291; doi:10.1136/bmjpo-2020-000799)
Supplement: Supplementary data [file bmjpo-2020-000799supp002.pdf]

Appendix II – SKPI Manual (English version)

1. Introduction

This semi-structured manual contains the instructions for the use of the Sexual Knowledge Picture Instrument (SKPI).

General aim is to look at each drawing in the picture book with the child, and ask the accompanying questions. Each interview should be video recorded, and given a scoring afterwards by the interviewer. The scoring pays attention to the general impressions, the verbal reactions and knowledge, and the non-verbal reactions of the child.

Children are normally open-minded and see almost everything on the drawings as normal. They will respond openly to the questions asked by the interviewer. If the child does not seem to want to tell something, this is remarkable.

It is therefore important to pay attention to:

- WHAT the child tells.

- HOW the child tells.

- IN WHAT CONTEXT the child tells it.

- What the child DOES NOT tell.

Before each interview, we recommend to read this manual and the picture book carefully, and to be aware of the instructions, notes and questions to ask with each picture.

1.1 General instructions to the child

At the start of the interview it is important to explain to the child what will happen, and what you expect from the child.

First give a short, neutral introduction:

"I have a booklet with drawings here."

"I'm going to show you all the drawings and then you can tell about it."

"I'm going to ask you some questions too."

"I'm going to record us on this small camera, so I can see what you told me once more."

Subsequently, make a number of things clear to the child:

1. Emphasize that as the interviewer you are "ignorant".

Therefore, please state that the child can NOT do it wrong, so there is no right or wrong answer to your questions (This is in contrast to for example situations at school, when the teacher asks a question).

Do this as follows:

"It's about what YOU know and what YOU want to tell me."

"Everything you say is always good. So you can't give wrong answers (like at school, if the teacher or teacher asks you something.) "

2. Don't know - instruction

"If you don't know the answer to the question, please say so."

Check whether the child has understood this, for example by asking the following question:

"So if I ask you, What's my dog's name? What do you say?" Possibly followed by: *“You can't know that, right, because I didn't tell you?”*

3. Don't understand - instruction

"If you don't get the question, you can just say that."

Check whether the child has understood this by asking the following questions:

"So if I ask you: What is your ‘gender’? What do you say then?” (the child probably responses something like: *“I don’t know/understand”*

"That's because gender is a difficult word. Then I will ask in another way, for example: are you a boy or a girl"?

Note: Recommended is to have a 2nd example ready in case the child guesses an answer.

1.2 General instructions for the interviewer

During the course of the SKPI-interview, follow the next instructions:

1. Ask open-ended questions (These questions are usually beginning with ‘what’, ‘where’, ‘who’, etc)

For example, *"What do you see here?"*

2. Do NOT ask suggestive questions

Those questions that lead or force the child to a certain 'expected' answer, such as: *"Did you do this with mummy too?"*

3. Avoid closed questions (that can only be answered with "yes" or "no")

Note 1: closed questions are not always suggestive. Sometimes even less than an open question, for example compare: *"Who did you discuss this with?" / "Did you discuss this?"*

Note 2: In case you feel it is necessary to help the child by giving multiple answer options, most young children tend to choose the last answer option. Take this into account.

4. Don't know, or don't want to tell?

If the child says nothing, or says he does not know, however, it seems to the researcher that the child does not WANT to tell it (for example, you notice this because the child says 'don't know' and looks away, clearly trying to distract the interviewer), then ask:

"Don't you know that, or don't you want to tell that?"

If the child answers "I don't want to tell you that", once ask why e.g.: *"Can you tell me why you don't want to tell this?"* Then respect the answer, and say *"okay"* and do not repeat the question.

5. Use the child's own words

During the conversation with the child, copy as much as possible from his / her words. For example, if the child will call the adults "mom and dad" in the drawings, or names the male genital "pee pee", continue using these same words throughout the rest of the interview.

6. Tell me more

Most young children still have a limited vocabulary, meaning they do not have the ability to express everything properly. They will therefore sometimes use their own, or different words for something. Therefore, at your own insight, ask more questions based on those given answers that might have a different meaning for the child.

E.g. at picture 14, when the child answers "That mother is washing the boy", ask *"Tell me, what's that, washing?"* and then *"Does your mother do that to you too?"*

7. Encourage

It is important to encourage the child every now and then, by saying things like *“You can really tell a lot / You do tell very clearly / You participate very well”*, etc.

Note: do not encourage the child by just saying something like *“That’s right”* (as there is no right or wrong in the child's answers).

8. React neutrally, even to remarkable statements

If the child gives a reaction that is striking to the interviewer, it is first of all important **to respond as normal / neutral as possible**. In addition, always respond briefly and by means of an open question, for example by asking (one or maximally two times):

"Tell me more ...?" Or, *"Can you tell me more about that?"*

After this, the child is free to tell more about this.

If the child tells more, ask open follow-up questions:

"And what happened then?" "And further?" Etc.

Only in case the child makes a fairly clear statement, ask more closed questions, such as:

"Who was that with?" and / or *"where was that exactly?"*

Then, ben careful to **always resume the interview in a normal manner**.

9. Ending the interview

After having looked at all the drawings, finish the interview by complimenting the child, and thank them for their efforts.

Then ask if he / she wants to say something else (which you have not asked or what has not been discussed). And finally ask if the child has any questions for you.

1.3 Video recording instructions

Ensure an easy set-up for the interview, preferable at a table, sitting next to the child. Use a small secured camera, preferably on a tripod.

Before starting the recording, make sure the child is clearly visible.

After finishing the interview do not forget to directly turn off the camera, and immediately store the recordings on a developed, secured database or at a secured server. Then, remove the recording from the camera.

Important note: remember to never leave this camera unattended, and always store it in a locker or other safe place after the interview.

2. The SKPI-interview

Read the following questions and notes carefully before each interview.

Look at each drawing with the child, and ask the questions from the scoring list below. Any relevant or striking statements made by the child should be noted directly. A complete scoring of the child's answers for each question can be given afterwards, based on the observation of the video recordings (chapter 4).

2.1 Picture 1: Introduction picture I (Family sitting at the table)

Aim: The child loosens up.

Questions (if necessary, so if the child does not tell spontaneously):

-*What do you see here?*

-*Who are they?*

-*Wat are they doing?*

Continue to ask until a short "story" was formulated by the child, e.g.: "A father and mother and child, they are eating."

Then encourage: "Do you know what you told me?" (Repeat what the child has said) "Well told!" or "Clearly told!"

2.2 Picture 2: Introduction picture II (Family with digital media / television)

Aim: The child loosens up.

Ask the following questions:

- *What do you see here?*

- *Who are they?*

- *Wat are they doing?*

Again, ask until a short story is told by the child, e.g. "Two children and their mom and dad sit on the couch watching television and playing games on the phone / laptop."

- Ask further about the TV / smartphone / laptop: *“Do you also have one at home? What do you see on it? / What can you do with that?”*

Eventually encourage the child again.

Note: In some (sexual) abuse of children imaging with smartphones takes place. Also, some children have been confronted with inappropriate sexual/pornographic or violent material. In those cases this picture may bring up other stories, and emotions in the child.

If the child's answer may be related to involuntary sexual or violent behavior (e.g. “I always see ‘grown-up’ television programs at grandpa’s home.”) ask: *“Tell me more”, or “Can you tell me tell you more about that?" and eventually ask more follow-up questions “where/how/when did you see this? Did it happen to you?”*

Then resume the interview in a normal manner.

2.3 Picture 3: Dressed children

Aim: Testing knowledge of gender differences, naming body parts

Ask the following questions:

- *What do you see?*

- *How do you know that's a boy and a girl?*

- *How do you see that?*

- *Tell me, why is someone is a boy or a girl?*

- *What are you, a boy or a girl?*

- *How do you know you're a boy / girl?*

2.4 Picture 4: Undressed children (front)

Aim: Testing knowledge of gender identity and naming genitals.

Ask the following questions:

-*What do you see?*

-*How do you know that's a boy and a girl?*

-By what can you tell that?

-Can you see it somewhere else?

Note: if the child does not spontaneously name the genitals, use a pencil to point out the genitals from both children, and ask: '*What's that?'* or *'How do you call that?'* Then resume the interview in a normal manner.

2.5 Picture 5: Undressed children (back)

Aim: Testing knowledge of body parts

Ask the following questions:

-What / who do you see?

Then use a pen to point out the buttocks from one of the children.

-What are those? And what can you do with it?

2.6 Picture 6: Dressed man and woman

Aim: Testing knowledge of body parts and their (different) functions.

Use a pen to point out the following body parts on the drawing: **eye, ear, mouth, hand, foot / leg.**

Meanwhile, ask the following questions:

-What is / are that?

-What can you do with that?

-Can you do anything else with it?

Note: Repeat the last question until the child does no longer name a function. Ask further if the child's answer can also be related to sexual behavior (e.g. is the child tells the function of the mouth is licking, or taking a bite; ask: *"Licking… of what? Can you tell me more about that"* Then resume the interview in a normal manner.

2.7 Picture 7: Undressed man and woman (front)

Aim: Testing knowledge of sexual body parts and their functions.

Again, use a pen to point out the following body parts : **female breasts, female genital, male genital.**

For each body part ask the following questions:

-*What is / are that?*

-*What can you do with that?*

-*Can you do anything else with it?*

Note: Ask further if the child's answers can also be related to sexual abusive behavior (e.g. if the child answers you can put a pencil into the female genital) ask: *"Can you tell me more about that?"* And: "*How do you know that, have you ever seen that?"*. If the child refers to urine, or urinating is mentioned in relation to the genital, ask what color this urine is, and if it can be a different color. If the answer to this is white, ask further: "*White transparent, like lemonade, or white like milk? Or more like yoghurt or like glue?*" And eventually ask: "*why do you think that/how do you know that, have you ever seen that?"* Then resume the interview in a normal manner.

2.8 Picture 8: Undressed man and woman (back)

Aim: Testing knowledge of body parts

Ask the following questions:

-*What / who do you see?*

-*What are those?*

-*What can you do with that? And can you do other things with it? Etc.*

2.9 Picture 9: Kissing man and woman

Aim: Testing knowledge of normal (fun) intimacy and voluntary (or eventually involuntary) sexual activities.

Ask the following questions:

- *What do you see here?*

- *Who are they?*

- *Wat are they doing?*

- *Can you tell me more?*

- *How do you think that mummy / woman feels about it? Why do you think so?*

- *How you think the daddy / father feels about it in the picture? Why do you think?*

- *Do you ever do that yourself? (if so: With who? / How do you like that?)*

Note: If the child's answer may be related to involuntary sexual behavior (e. g. *"Daddy is going to undress Mommy, he wants to make love to her but she doesn't want to."*) ask: *"Tell me more"*, or *"Can you tell me tell you more about that?"* and eventually ask more follow-up questions *"where/how/when did you see this? Did it happen to you?"* Then resume the interview in a normal manner.

2.10 Picture 10: Man and woman being intimate

Aim: Testing knowledge of normal (fun) intimacy and voluntary (or eventually involuntary) sexual behavior.

Ask the following questions:

- *What do you see here?*

- *Who are they?*

- *Wat are they doing?*

- *Can you tell me more?*

- *How do you think the lady / mama feels about it? Why do you think so / how do you know that?*

- *How does the mister / dad feels about it? How do you know that?*

Note: If the child's answer may be related to non-voluntary sexual behavior (e.g. "Daddy is peeing on me.") ask further: *"Tell me more about that…"*. Eventually, ask follow-up questions, such as: *"Did this happen to you, or have you seen it with someone?"* and/ or *"What happened exactly?" "Can you tell me more about that?"* Then resume the interview in a normal manner.

2.11 Picture 11: Undressed children's play

Aim: Testing knowledge of normal (fun) intimacy and voluntary sexual behavior.

Ask the following questions:

- *What do you see here?*

- *Who are they?*

- *Wat are they doing?*

- *Can you tell me more?*

- *How do you think the girl feels about it? Why do you think?* Or: *How do you know she likes it/ feels happy about it?*

- *How do you think that boy feels about it? Why do you think?*

- *Do you ever do this yourself?* If so: *How do you feel about that?*

Beware: If the child's answer may refer to with sexual and/or abusive behavior (e.g. "Ally should not do that, no one should touch my pee pee.") ask further: *"Tell me about that"* or *"Can you tell me more about that?"* and eventually ask further: *"Have you ever experienced that? Yourself or have you seen it with someone? What happened?"* Then resume the interview in a normal manner.

2.12. Picture 12: Child with a doctor figure

Aim: Testing knowledge of normal intimacy and voluntary sexual behavior.

Ask the following questions:

- *What do you see here?*

- *Who are they?*

- *Wat are they doing?*

- *Can you tell me more?*

- *What could be going on? / What happened?*

- *How do you think that daddy / doctor feels about it? Why do you think so?*

- *How you think the baby feels about it in the picture? Why do you think so?*

Note: If the child's answer may refer to sexual and/or abusive behavior (e.g. "That doctor should not do that, no one should touch my pee pee.") ask: "*Tell me about that"* or *"Can you tell me more about that?"* and eventually ask further: *"Have you ever experienced that? Yourself or have you seen it with someone? What happened?"* Then resume the interview in a normal manner.

2.13 Picture 13: Father figure bent over girl in bed

Aim: Testing knowledge of normal intimacy and voluntary sexual behavior.

Ask the following questions:

- What do you see here?

- Who are they?

- Wat are they doing?

- Can you tell me more?

- How do you think the girl / child feels about it? Why do you think so?

- How do you think the father / grandpa / man feels about it? Why do you think so?

- Do you ever do this yourself? How do you feel about it?

Note: If the child's answer may refer to sexual and/or abusive behavior (e.g. “Grandpa always does that in the middle of the night.”) ask further: "*Tell me about that*" or *"Can you tell me more about that?”* and eventually ask further: *“Have you ever experienced that? Yourself, or have you seen it with someone? What happened?”* Then resume the interview in a normal manner.

2.14 Picture 14: Naked boy in the shower with mother figure

Aim: Testing knowledge of normal intimacy and (in)voluntary sexual behavior.

Ask the following questions:

- What do you see here?

- Who are they?

- Wat are they doing?

- Can you tell me more?

- How do you think the boy / child feels about it? Why do you think so?

- How do you think the mother / woman feels about it? Why do you think so?

- Do you ever do this? How do you feel about it?

Note: If the child's answer may refer to with sexual and/or abusive behavior (e.g. “That washing hurts!”) ask: "*Tell me about that*" or "*Can you tell me more about that?”* and eventually ask further: *“Have you ever experienced that? Yourself, or have you seen it with someone? What happened?”* Then resume the interview in a normal manner.

2.15 Picture 15: Mother figure with crying child in bed

Aim: Testing knowledge of normal intimacy and (in)voluntary sexual behavior.

Ask the following questions:

- *What do you see here?*

- *Who are they?*

- *Wat are they doing?*

- *Can you tell me more?*

- *How do you think the boy / child feels about it? Why do you think so?*

- *How do you think the mother / woman feels about it? Why do you think so?*

- *Do you ever do this? How do you feel about it?*

Notes: If the child refers to having a nightmare / bad dream, or being hurt ask further: "Tell me about that" or "Can you tell me more about that?" and eventually ask further: "Do you have nightmares yourself? Can you tell me more about what happens?"

If the answer may refer to with sexual and/or abusive behavior (e.g. child tells about a related dream, or that the mother figure touches the child in a way he / she does not want) ask: "*Tell me about that*" or "*Can you tell me more about that?"* and eventually ask further: *"Have you ever experienced that? Yourself, or have you seen it with someone? What happened?"* Then resume the interview in a normal manner.

3. General impressions and remarks

During the interview the child was:

□ open / open-minded

□ loaded / fraught

Verbal remarks (including wonderful associations):

Non-verbal remarks (including noticeable behavioral changes):

Other notable impressions of the child / situations during the interview:

4. Verbal knowledge scoring

Scoring instructions:

If a child answers

If a child answers "I don't know" or "Just because",

- In case of questions testing the knowledge score as NO / NOT GOOD.
- When the child is asked to judge the situation on the picture, or feeling of the character, score as OTHERWISE, NAMELY… and write "I don't know".

If a question from the manual was not asked, then score as OTHERWISE, NAMELY…, and then fill in "not asked". - For repeated questions such as gender identity / genitalia functions, score again and again, do not include the score from previous questions.

If two answers are given, of which one is right and one is wrong (for example with the male genital function: pooping and peeing), count this as GOOD.

Always tick only 1 answer (which is closest). Multiple answers cannot be processed.

If the answer is not said, but is clearly portrayed by the child (for example, if function of hands is asked, and the child claps), count this as GOOD.

Verbal scoring list:

Picture 3: Dressed children

Difference between boy and girl

□ no

□ yes

□ doesn’t want to tell

□ otherwise, namely….

Motivation

□ none (child gives no motivation)

□ yes, cultural differences (e.g. clothing, long hair)

□ yes, genital differences

□ doesn’t want to tell

□ otherwise, namely….

Own gender

□ no

□ yes

□ doesn’t want to tell

□ otherwise, namely….

Motivation

□ none (child gives no motivation)

□ yes, cultural differences (e.g. I'm playing football, I have a ponytail)

□ yes, genital differences

□ doesn’t want to tell

□ otherwise, namely….

Picture 4: Undressed children (front)

Difference between boy and girl

□ no

□ yes

□ doesn’t want to tell

□ otherwise, namely….

Motivation

□ none (child gives no motivation)

□ yes, cultural differences (e.g. clothing, long hair)

□ yes, genital differences

□ doesn’t want to tell

□ otherwise, namely….

Knowledge female genital

□ no

□ yes (count all terms correct: from pee or pussy to butterfly)

□ doesn’t want to tell

□ otherwise, namely….

Function female genital

□ not good (says nothing relevant)

□ good (only functional : usually peeing)

□ good (incl sexual function), namely….

□ doesn’t want to tell

□ otherwise, namely….

Knowledge male genital

□ no

□ yes (all words correct: penis, wee wee, willy etc.)

□ doesn’t want to tell

□ otherwise, namely….

Male genital function

□ no good answer

□ good (only functional: usually urinating)

□ good (incl sexual function), namely….

□ doesn't want to tell

□ otherwise, namely….

Picture 5: Undressed children (back)

Knowledge buttocks

□ no

□ yes (all words correct: bibs, butt, etc)

□ doesn't want to tell

□ otherwise, namely….

Buttocks function

□ not good

□ good (if at least one function, usually sitting or pooping)

□ doesn't want to tell

□ otherwise, namely….

Picture 6: Dressed man and woman

Knowledge eyes

□ no

□ yes

□ doesn’t want to tell

□ otherwise, namely….

Eyes function

□ no good answer

□ good (as one good function: seeing, looking, blinking, staring, etc)

□ will not tell

□ otherwise, namely….

Knowledge ears

□ no

□ yes

□ doesn’t want to tell

□ otherwise, namely….

Ears function

□ no good answer

□ good (as one good function: hearing, listening, etc)

□ doesn’t want to tell

□ otherwise, namely….

Knowledge lips / mouth

□ no (says no lips and no mouth)

□ yes (says lips and / or mouth)

□ doesn’t want to tell

□ otherwise, namely….

Function lips / mouth

□ not good (does not say anything relevant)

□ good (as one good function: talking, eating, yawning, putting on lipstick, licking, eating, etc)

□ doesn’t want to tell

□ otherwise, namely….

Knowledge hands

□ no

□ yes

□ doesn’t want to tell

□ otherwise, namely….

Hands function

□ not good

□ good (as soon as one good function: grab, clap, tickle, etc)

□ doesn’t want to tell

□ otherwise, namely….

Knowledge legs / feet

□ no

□ yes

□ doesn’t want to tell

□ otherwise, namely….

Leg / feet function

□ not good

□ good (as soon as one good function: running, running, etc)

□ doesn’t want to tell

□ otherwise, namely….

Picture 7: Undressed man and woman (front)

Knowledge breasts

□ no

□ yes (all words right: breasts, tits, boobies, etc)

□ doesn’t want to tell

□ otherwise, namely….

Breast function

□ not good (says nothing relevant)

□ good (as one good function: for the baby to drink milk, eat for the baby, etc)

□ doesn’t want to tell

□ otherwise, namely….

Female genital knowledge

□ no

□ yes (all words correct: pussy, poeni, pee-hole, etc)

□ doesn’t want to tell

□ otherwise, namely….

Female genital function

□ not good (does not say anything relevant)

□ good (only functional :: usually urinate)

□ good (incl sexual function), namely….

□ doesn’t want to tell

□ otherwise, namely….

Knowledge male genital

□ no

□ yes (all words correct: wee wee, pee pee, etc)

□ doesn't want to tell

□ otherwise, namely….

Male genital function

□ not good

□ good (function only: usually peeing)

□ good (incl sexual function), namely…

□ doesn't want to tell

□ otherwise, namely….

Questioning the color of the puddle:

□ yellow / white (transparent), or other logical answer

□ white (as in milk / glue / yogurt, etc)

□ doesn't want to tell

□ otherwise, namely…

Picture 8: Undressed man and woman (back)

Knowledge buttocks

□ no

□ yes (all words correct: ass, butt, etc)

□ doesn't want to tell

□ otherwise, namely….

Buttocks function

□ not good

□ good (as soon as one good function: usually sitting or defecating)

□ doesn’t want to tell

□ otherwise, namely….

Picture 9: Kissing man and woman

Knowledge (child shows basic insight in situation)

□ no

□ yes (as soon as something like kissing / hugging, hugging is in the answer)

□ doesn’t want to tell

□ otherwise, namely….

Estimated feeling female figure

□ not nice

□ nice

□ doesn’t want to tell

□ otherwise, namely….

Estimated feeling male figure

□ not nice

□ nice

□ doesn’t want to tell

□ otherwise, namely….

Picture 10: Man and woman being intimate

Knowledge (child shows basic insight in situation)

□ no (child describes what he / she sees, without insight, for example they lie (naked) on / look at each other)

□ yes (as soon as something like kissing / hugging, hugging, sex is in the answer)

□ doesn’t want to tell

□ otherwise, namely….

Estimated feeling female figure

□ not nice

□ nice

□ doesn’t want to tell

□ otherwise, namely….

Estimated feeling male figure

□ not nice

□ nice

□ doesn’t want to tell

□ otherwise, namely….

Picture 11: Undressed children’s play

Knowledge (child shows basic insight in situation)

□ no

□ yes (if something like touching / looking / pointing / playing, or normal curiosity about the other is in the answer.

□ doesn’t want to tell

□ otherwise, namely….

Estimated feeling girl figure

□ not nice

□ nice

□ doesn’t want to tell

□ otherwise, namely….

Estimated feeling boy figure

□ not nice

□ nice

□ doesn’t want to tell

□ otherwise, namely….

Picture 12: Child with a doctor figure

Knowledge (child shows basic insight in the situation)

□ no (child describes what he / she sees, without insight, for example no doctor or father, but someone who has hands there)

□ yes (as soon as the doctor answers the question, father changing or changing a child)

□ doesn’t want to tell

□ otherwise, namely….

Estimated feeling doctor figure

□ nice

□ nice

□ doesn’t want to tell

□ otherwise, namely….

Estimated feeling child figure

□ not nice

□ nice

□ doesn’t want to tell

□ otherwise, namely….

Picture 13: Father figure bent over girl in bed

Knowledge (child shows basic insight in the situation)

□ no (child describes what he / she sees, without insight, e.g. goes to sleep, or lies in bed, but does not tell what father does, even when asked)

□ yes (e.g. if saying good night before bed, waking up to go to school is in the answer)

□ doesn’t want to tell

□ otherwise, namely… (e.g. also if child says sleeping, but no further question is asked about what father does)

Estimated feeling male figure

□ not nice

□ nice

□ doesn’t want to tell

□ otherwise, namely….

Estimated feeling child figure

□ not nice

□ nice

□ doesn’t want to tell

□ otherwise, namely….

Picture 14: Naked boy in the shower with mother figure

Knowledge (child shows basic insight in the situation)

□ no (child describes what he / she sees, without insight, eg child in the shower, but does not know what the mother does when asked)

□ yes (if something is washed from child by mother is in the answer))

□ doesn’t want to tell

□ otherwise, namely…. (eg child says shower, but is not asked what mother does)

Estimated feeling female figure

□ not nice

□ nice

□ doesn’t want to tell

□ otherwise, namely….

Estimated feeling child figure

□ not nice

□ nice

□ doesn’t want to tell

□ otherwise, namely….

Picture 15: Mother figure with crying child in bed

Knowledge (child shows basic insight in the situation)

□ no (child describes what he / she sees, without insight, eg child cries, but does not know (when inquiring) why)

□ yes (as soon as something has been dreamed of, not being able to sleep, being in pain)

□ doesn’t want to tell

□ otherwise, namely…. (eg, child says the baby is crying, but it is not asked why it is crying)

Estimated feeling female figure

□ not nice

□ nice

□ doesn’t want to tell

□ otherwise, namely….

Estimated feeling child figure

□ not nice

□ nice

□ doesn’t want to tell

□ otherwise, namely….

5. Scoring nonverbal responses

The following table can be used to score the child's nonverbal responses for each picture. If other non-verbal responses are noticed during the interview, these can be written down (and eventually explained) at the bottom of the table.

	Nr	**1**	2	3	4	5	6	7	8	9	10	11	12	13	14	15
Being silent / not want to say anything	1															
Speak with a soft(er) voice	2															
Speak with a different (weird or louder) voice	3															
Giggling	4															
Looking at the picture with disgust	5															
Looking at the picture with a fearful or frozen watch	6															

Turning the page / trying to continue to the next picture	7															
Trying to close the book	8															
Walking away (to parents / other room)	9															
Going to the toilet	10															
Looking away from the picture / not willing to look at the picture	11															
Avoiding eye contact with the interviewer	12															
Extracting the interviewer (telling irrelevant story, playing, etc)	13															
Showing weird faces	14															
Looking sad / depressed	15															
Crying	16															
Looking angry	17															
Sitting with hunched shoulders / crouched down	18															

Putting hands in front of the mouth (e.g. as an expression of surprise or disbelief)	19															
Putting your hands over the eyes	20															
Hiding head / face in clothes	21															
Putting head on arms or on table	22															
Not being able to sit still / wobble constantly	23															
No scoring (because of not wanting to participate in the study at all anymore.)	24															
Otherwise, namely…	25															
Otherwise, namely	26															
