# Supplementary material for: Validation of the Sexual Knowledge Picture Instrument as a diagnostic instrument for child sexual abuse: study protocol
Source: BMJ Paediatr Open. 2020 Sep 29;4(1):e000799. doi: 10.1136/bmjpo-2020-000799 (PMC7526291; doi:10.1136/bmjpo-2020-000799)
Supplement: Supplementary data [file bmjpo-2020-000799supp003.pdf]

Vragenlijsten Ouder / Verzorger

PICAS Studie

Onderzoeker: _ _ _ _ _ _ _ _ _ _ _ _ _ _ _ *(in te vullen door de onderzoeker)*

Onderzoeksnummer: _ _ _ _ _ _ _ _ _ _ _ *(in te vullen door de onderzoeker)*

Datum onderzoek: _ _ - _ _ - _____ *(in te vullen door de onderzoeker)*

Indien de onderzoeker tijdens het onderzoek van uw kind bijzonderheden vindt, zal er contact worden gezocht met u. Wij vragen u daarom hieronder contactgegevens in te vullen van u / uw partner. Deze pagina zal los van de overige pagina's van de vragenlijst worden bewaard.

Bereikbaar via telefoonnummer:

- ☐ Moeder ....
- ☐ Vader ....
- ☐ Anders, namelijk..

Emailadres (eventueel): ....

Deze vragenlijst is ingevuld door (één antwoord mogelijk, kruis svp het juiste antwoord aan):

- ☐ (Biologische) moeder
- ☐ (Biologische) vader
- ☐ Pleegvader
- ☐ Pleegmoeder
- ☐ Voogd
- ☐ Anders, namelijk...

Supplemental material BMJ Publishing Group Limited (BMJ) disclaims all liability and responsibility arising from any reliance placed on this supplemental material which has been supplied by the author(s) BMJ Paediatrics Open

GEGEVENS OVER HET KIND

1. **Wat is de geboortedatum van het kind?** _ _ /_ _ / _ _ _ _

2. **Bij wie woont het kind op dit moment?**

- ☐ Bij beide biologische ouders (samen)
- ☐ Bij beide biologische ouders (apart)
- ☐ Bij één van de biologische ouders, namelijk..............................
- ☐ Anders, namelijk..

3. **In welk land is het kind geboren?**

....

4. **Welke taal spreekt het kind het meest?**

- ☐ Nederlands
- ☐ Turks
- ☐ Arabisch
- ☐ Anders, namelijk..

5. **Is er daarnaast een tweede taal die het kind (goed) beheerst?**

- ☐ Nee
- ☐ Ja, namelijk...

6. **Naar wat voor school gaat het kind?**

☐ Voorschool

☐ Reguliere (basis)school

☐ Speciaal onderwijs, namelijk.............................

☐ Anders, namelijk.............................

Supplemental material BMJ Publishing Group Limited (BMJ) disclaims all liability and responsibility arising from any reliance placed on this supplemental material which has been supplied by the author(s) BMJ Paediatrics Open

GEGEVENS OVER DE BIOLOGISCHE OUDER(S)

Onderstaande vragen gaan over de biologische ouders van het kind.

(Als u zelf een van de biologische ouders bent vul hier dan uw eigen situatie in)

7. **Wat is het geboorteland van de biologische vader van het kind?**

....

8. **Wat is het geboorteland van <u>de ouders van</u> de biologische vader van het kind?**

....

9. **Wat is het geboorteland van de biologische moeder van het kind?**

....

10. **Wat is het geboorteland van <u>de ouders van</u> de biologische moeder van het kind?**

....

van Ham K, *et al. BMJ Paediatrics Open* 2020; 4:e000799. doi: 10.1136/bmjpo-2020-000799

Supplemental material BMJ Publishing Group Limited (BMJ) disclaims all liability and responsibility arising from any reliance placed on this supplemental material which has been supplied by the author(s) BMJ Paediatrics Open

VOORGESCHIEDENIS EN BETROKKENHEID INSTANTIES

6. Is het kind nu of in het verleden (voor zover u weet)

	Ja	Nee	Mogelijk / onbekend
a. Bekend met een verstandelijke beperking?			
b. Bekend met ernstige problemen met het zien of horen?			
c. Bekend met een gedragsprobleem?			
d. Slachtoffer (geweest) van seksueel misbruik?			
e. Slachtoffer (geweest) van een andere vorm van kindermishandeling?			
f. Getuige (geweest) van huiselijk geweld?			
g. Behandeld door een psycholoog of psychiater?			
h. Indien ja bij g.: voor welke diagnose is hij/zij behandeld?			

7. Is er bij het kind, nu of in het verleden (voor zover u weet)

	Ja	Nee	Mogelijk / onbekend
a. Betrokkenheid (geweest) van Veilig Thuis (voorheen Advies en Meldpunt Kindermishandeling, of AMK)?			
b. Betrokkenheid (geweest) van Jeugdzorg / JBRA?			
c. Betrokkenheid (geweest) van de Raad voor Kinderbescherming?			
d. Betrokkenheid (geweest) van de (zeden)politie?			

van Ham K, et al. BMJ Paediatrics Open 2020; 4:e000799. doi: 10.1136/bmjpo-2020-000799

Supplemental material
BMJ Publishing Group Limited (BMJ) disclaims all liability and responsibility arising from any reliance
placed on this supplemental material which has been supplied by the author(s)
BMJ Paediatrics Open

**e. Betrokkenheid (geweest) van een andere instantie, namelijk…………………..**			

- Einde van de vragenlijst. Hartelijk dank voor uw medewerking! -

----

van Ham K, *et al. BMJ Paediatrics Open* 2020; 4:e000799. doi: 10.1136/bmjpo-2020-000799

CSBI

Vragenlijst

Adapted and reproduced by special permission of the Publisher, Psychological Assessment Resources, Inc., 16204 North Florida Avenue, Lutz, Florida 33549, from the Child Sexual Behavior Inventory by William Friedrich, Ph.D., Copyright 1986, 1991, 1992, 1993, 1997 by Mayo Clinic Foundation, Inc. Further reproduction is prohibited without permission of Psychological Assessment Resources, Inc.

Naam van het kind____

Geslacht (omcirkel): jongen meisje

Leeftijd van het kind____

Geboortedatum van het kind______________________________________

Uw naam____

Uw relatie tot het kind____

Datum van vandaag____

Instructies

Deze vragenlijst stelt vragen over het gedrag van uw kind. Lees elke vraag zorgvuldig en omcirkel vervolgens het cijfer dat weergeeft hoe vaak uw kind het gedrag heeft laten zien in de afgelopen 6 maanden.

Omcirkel **0** als uw kind het gedrag **nooit** heeft laten zien	(0)	1	2	3
Omcirkel **1** als uw kind het gedrag **minder dan eens per maand** heeft laten zien	0	(1)	2	3
Omcirkel **2** als uw kind het gedrag **1-3 keer per maand** heeft laten zien	0	1	(2)	3
Omcirkel **3** als uw kind het gedrag **tenminste een keer per week** heeft laten zien	0	1	2	(3)

Bijvoorbeeld, als uw kind ongeveer twee keer per maand te laat komt op school, dient u het cijfer 2 te omcirkelen voor deze vraag, zoals hieronder:

Komt te laat op school	0	1	(2)	3

Als u een fout heeft gemaakt of uw antwoord wilt wijzigen, gelieve NIET te wissen. Doorkruis het foute antwoord met een "X" en omcirkel vervolgens het juiste antwoord, zoals hieronder:

Komt te laat op school	0	(1)	(2) X	3

Vragen over het gedrag van uw kind

Omcirkel het cijfer dat weergeeft hoe vaak uw kind de onderstaande gedragingen heeft laten zien in de afgelopen 6 maanden:

		Nooit	Minder dan 1 maal per maand	1-3 maal per maand	Minstens 1 maal per week
1.	Verkleedt zich als een persoon van het andere geslacht	0	1	2	3
2.	Staat te dicht op andere mensen	0	1	2	3
3.	Zegt van het andere geslacht te willen zijn	0	1	2	3
4.	Raakt zijn/haar geslachtsdelen aan in het openbaar	0	1	2	3
5.	Masturbeert met de hand	0	1	2	3
6.	Tekent kinderen en/of volwassenen met geslachtsdelen en borsten	0	1	2	3
7.	Raakt de borsten van moeder of andere vrouw aan of probeert dit te doen	0	1	2	3
8.	Masturbeert met een voorwerp (deken, kussen, speelgoed)	0	1	2	3
9.	Raakt de geslachtsdelen van andere kinderen aan	0	1	2	3
10.	Probeert de geslachtsgemeenschap na te doen met een kind of volwassene	0	1	2	3
11.	Komt met mond aan geslachtsdelen van andere kinderen of volwassenen	0	1	2	3
12.	Raakt thuis zijn/haar geslachtsdelen aan	0	1	2	3
13.	Raakt geslachtsdelen van volwassenen aan	0	1	2	3
14.	Raakt geslachtsdelen van dieren aan	0	1	2	3
15.	Maakt seksuele geluiden (zuchten, luidruchtig ademhalen, hijgen…)	0	1	2	3
16.	Vraagt aan anderen om met hem/haar seksuele handelingen uit te voeren	0	1	2	3
17.	Wrijft met het lichaam tegen andere mensen of meubelen	0	1	2	3
18.	Brengt voorwerpen in haar vagina of zijn/haar anus	0	1	2	3
19.	Probeert naar mensen te kijken als zij naakt zijn of zich aankleden	0	1	2	3
20.	Doet alsof poppen of (pluche) dieren seks hebben	0	1	2	3

BMJ Publishing Group Limited (BMJ) disclaims all liability and responsibility arising from any reliance placed on this supplemental material which has been supplied by the author(s)

21.	Toont zijn/haar geslachtsdelen aan volwassenen	0	1	2	3
22.	Probeert afbeeldingen te bekijken van (half)naakte mensen (bijv. in tijdschriften)	0	1	2	3
23.	Praat over seksuele handelingen	0	1	2	3
24.	Kust volwassenen die hij/zij niet goed kent	0	1	2	3
25.	Raakt van streek als volwassenen elkaar kussen of omhelzen	0	1	2	3
26.	Is overdreven vriendelijk tegen mannen die hij/zij niet goed kent	0	1	2	3
27.	Kust kinderen die hij/zij niet goed kent	0	1	2	3
28.	Praat op een flirtende manier	0	1	2	3
29.	Probeert andere kinderen tegen hun zin uit te kleden (blouse, broek open doen…)	0	1	2	3
30.	Wil televisie programma's of films zien waar naakt of seks in voorkomt	0	1	2	3
31.	Probeert te tongzoenen bij het kussen	0	1	2	3
32.	Omhelst (knuffelt) volwassenen die hij/zij niet goed kent	0	1	2	3
33.	Toont zijn/haar geslachtsdelen aan andere kinderen	0	1	2	3
34.	Probeert volwassenen tegen hun zin uit te kleden (blouse, broek open doen…)	0	1	2	3
35.	Heeft sterke interesse voor het andere geslacht	0	1	2	3
36.	Raakt met zijn/haar mond de borsten van moeder of een andere vrouw aan	0	1	2	3
37.	Weet meer over seks dan andere kinderen van zijn/haar leeftijd	0	1	2	3
38.	Ander seksueel gedrag: graag beschrijven<br>A. ………………………………………………………………………………<br>B. ………………………………………………………………………………	0	1	2	3

De volgende onderwerpen hebben te maken met wat kinderen doen, voelen of ervaren. Wilt u aangeven hoe vaak uw kind (het kind) elk van de volgende dingen heeft gedaan of ervaren **in de afgelopen maand.**

		Niet	Soms	Vaak	Heel vaak
1)	Driftbuien	1	2	3	4
2)	Lijkt droevig	1	2	3	4
3)	Liegen	1	2	3	4
4)	Heeft nare dromen of nachtmerries	1	2	3	4
5)	Leeft in een fantasiewereld	1	2	3	4
6)	Lijkt meer over seks te weten dan hij/zij zou behoren te weten	1	2	3	4
7)	Is snel bang	1	2	3	4
8)	Wil niet naar plaatsen gaan die hem/haar aan het verleden herinneren	1	2	3	4
9)	Is bang dat zijn/haar eten vergiftigd is	1	2	3	4
10)	Deinst terug of schrikt wanneer iemand snel beweegt of bij een hard geluid	1	2	3	4
11)	Heeft last van herinneringen aan iets wat hem/haar is overkomen	1	2	3	4
12)	Is bang dat iemand seksueel contact met hem/haar zal hebben	1	2	3	4
13)	Wil niet praten over wat er met hem/haar is gebeurd	1	2	3	4
14)	Doet dingen niet die hij/zij wel behoort te doen	1	2	3	4
15)	Maakt met opzet dingen stuk	1	2	3	4
16)	Praat over seksuele dingen	1	2	3	4
17)	Heeft problemen met de concentratie	1	2	3	4
18)	Geeft zichzelf de schuld voor dingen die niet zijn/haar schuld zijn	1	2	3	4
19)	Is bang wanneer hij/zij wordt herinnerd aan iets wat in het verleden is gebeurd	1	2	3	4
20)	Doet net alsof hij/zij geslachtsgemeenschap heeft	1	2	3	4
21)	Maakt zich zorgen dat er in de toekomst vervelende dingen zullen gebeuren	1	2	3	4
22)	Ruzie maken	1	2	3	4

BMJ Publishing Group Limited (BMJ) disclaims all liability and responsibility arising from any reliance placed on this supplemental material which has been supplied by the author(s)

		Niet	Soms	Vaak	Heel vaak
23)	Vechten	1	2	3	4
24)	Maakt tekeningen over iets vervelends wat er met hem/haar is gebeurd	1	2	3	4
25)	Heeft niet in de gaten wat hij/zij aan het doen was	1	2	3	4
26)	Heeft moeite met stilzitten	1	2	3	4
27)	Speelt over iets vervelends dat echt met hem/haar is gebeurd	1	2	3	4
28)	Lijkt versuft	1	2	3	4
29)	Heeft moeite om zich een vervelende gebeurtenis die in het verleden is gebeurd te herinneren	1	2	3	4
30)	Drugsgebruik	1	2	3	4
31)	Is bang in het donker	1	2	3	4
32)	Is bang om alleen te zijn	1	2	3	4
33)	Wegdromen	1	2	3	4
34)	Is erg agressief	1	2	3	4
35)	Raakt geslachtsdelen aan van andere kinderen of volwassenen (onder of boven de kleren)	1	2	3	4
36)	Ziet, voelt of hoort opeens iets vervelends dat in het verleden is gebeurd	1	2	3	4
37)	Hoort stemmen die hem/haar vertellen iemand iets aan te doen	1	2	3	4
38)	Staart voor zich uit	1	2	3	4
39)	Verandert van onderwerp of geeft geen antwoord als er iets gevraagd wordt over iets vervelends dat met hem/haar is gebeurd	1	2	3	4
40)	Heeft zenuwinzinkingen	1	2	3	4
41)	Lacht niet/is niet gelukkig als andere kinderen	1	2	3	4
42)	Huilt 's nachts omdat hij/zij bang is	1	2	3	4
43)	Slaat volwassenen (ouders)	1	2	3	4
44)	Is bang voor mannen	1	2	3	4
45)	Kan niet opletten	1	2	3	4
46)	Lijkt mijlenver weg te zijn	1	2	3	4

		Niet	Soms	Vaak	Heel vaak
47)	Schrikt snel	1	2	3	4
48)	Is op zijn/haar hoede voor mogelijk gevaar	1	2	3	4
49)	Doet geen dingen meer waar hij/zij vroeger van kon genieten	1	2	3	4
50)	Wordt bang of is verontrust wanneer hij/zij iets seksueels hoort of ziet	1	2	3	4
51)	Slaapt twee dagen of langer niet	1	2	3	4
52)	Let niet op omdat hij/zij in de eigen wereld is	1	2	3	4
53)	Maakt fouten	1	2	3	4
54)	Huilt zonder duidelijke reden	1	2	3	4
55)	Wil niet in de buurt zijn van iemand die iets vervelends bij hem/haar heeft gedaan of die hem/haar aan iets vervelends herinnert	1	2	3	4
56)	Is gespannen	1	2	3	4
57)	Maakt zich zorgen om de veiligheid van anderen	1	2	3	4
58)	Wordt erg boos om iets kleins	1	2	3	4
59)	Tekent seksuele dingen	1	2	3	4
60)	Trekt zijn/haar haren uit	1	2	3	4
61)	Noemt zichzelf slecht, dom of lelijk	1	2	3	4
62)	Gooit dingen naar vrienden of familieleden	1	2	3	4
63)	Raakt overstuur door iets uit het verleden	1	2	3	4
64)	Is tijdelijk blind of verlamd (geweest)	1	2	3	4
65)	Raakt overstuur door iets seksueels	1	2	3	4
66)	Gaat 's avonds niet meteen naar bed als dat (voor de 1e keer) gevraagd wordt	1	2	3	4
67)	Bang dat hij/zij door iemand vermoord wordt	1	2	3	4
68)	Zegt dat niemand hem/haar aardig vindt	1	2	3	4
69)	Huilt wanneer hij/zij wordt herinnerd aan iets uit het verleden	1	2	3	4
70)	Zegt dat iets vervelends hem/haar ***niet*** is gebeurd, ook al is dat wel zo	1	2	3	4

BMJ Publishing Group Limited (BMJ) disclaims all liability and responsibility arising from any reliance placed on this supplemental material which has been supplied by the author(s)

		Niet	Soms	Vaak	Heel vaak
71)	Zegt dat hij/zij dood wil gaanof doodgemaakt wil worden	1	2	3	4
72)	Doet alsof hij/zij geen gevoelens heeft bij het vervelende dat hem/haar is overkomen	1	2	3	4
73)	Zeurt	1	2	3	4
74)	Slaapt niet goed	1	2	3	4
75)	Maakt zich zorgen over seksuele dingen	1	2	3	4
76)	Wordt bang voor dingen waarvoor hij/zij vroeger niet bang voor was	1	2	3	4
77)	Hallucineert	1	2	3	4
78)	Gedraagt zich alsof hij/zij in een trance is	1	2	3	4
79)	Vergeet zijn/haar eigen naam	1	2	3	4
80)	Raakt overstuur als hij/zij herinnerd wordt aan het vervelende dat in het verleden is gebeurd	1	2	3	4
81)	Ontwijkt dingen die hem/haar herinneren aan het vervelende dat in het verleden is gebeurd	1	2	3	4
82)	Is lichtgeraakt	1	2	3	4
83)	Maakt rommel	1	2	3	4
84)	Lijkt verdrietig of depressief	1	2	3	4
85)	Is zo afwezig dat hij/zij niet opmerkt wat er om hem/haar heen gebeurt	1	2	3	4
86)	Wil bepaald voedsel niet eten	1	2	3	4
87)	Schreeuwt tegen familie, vrienden of leerkrachten	1	2	3	4
88)	Speelt niet omdat hij/zij depressief is	1	2	3	4
89)	Is ongehoorzaam	1	2	3	4
90)	Doet met opzet andere kinderen of familieleden pijn	1	2	3	4
